# Polypharmacy Is Associated with Sociodemographic Factors and Socioeconomic Status in United States Adults

**DOI:** 10.3390/pharmacy12020049

**Published:** 2024-03-12

**Authors:** Vishal Vennu

**Affiliations:** Department of Rehabilitation Sciences, College of Applied Medical Sciences, King Saud University, P.O. Box 10219, Riyadh 11433, Saudi Arabia; vvennu@ksu.edu.sa; Tel.: +966-114698770

**Keywords:** polypharmacy, socioeconomic status, United States

## Abstract

A thorough understanding of polypharmacy is required to create public health initiatives that minimize the potential for adverse outcomes. This study aimed to investigate the relationship between sociodemographic factors, socioeconomic status (SES), and polypharmacy risk in United States (US) individuals between 1999–2000 and 2017–2018. The cross-sectional National Health and Nutrition Examination Survey dataset covered ten cycles between 1999–2000 and 2017–2018. All individuals aged ≥18 years were included. The simultaneous use of at least five medications by one person is known as polypharmacy. Multivariable logistic regression showed that there was a statistically significant association between polypharmacy sociodemographic factors (such as age between 45 and 64 (odds ratio [OR] = 3.76; 95% confidence interval [CI] = 3.60–3.92; *p* < *0.0001*) and age of 65 years or above (OR = 3.96; 95% CI = 3.79–4.13; *p* < *0.0001*), especially women (OR = 1.09; 95% CI = 1.06–1.13; *p* < *0.0001*), non-Hispanic blacks (OR = 1.66; 95% CI = 1.51–1.83; *p* < *0.0001*), and veterans (OR = 1.27; 95% CI = 1.22–1.31; *p* < *0.0001*)) and SES (such as being married (OR = 1.14; 95% CI = 1.08–1.19; *p = 0.031*), widowed, divorced, or separated (OR = 1.21; 95% CI = 1.15–1.26; *p* < *0.0001*), a college graduate or above (OR = 1.21, 95% CI = 1.15–1.27, *p* < *0.0001*), and earning > USD 55,000 per year (OR = 1.86; 95% CI = 1.79–1.93; *p* < *0.0001*)). Individuals aged 45 years and above, women, and non-Hispanic blacks with higher educational levels and yearly incomes were more likely to experience polypharmacy in the US between 1999–2000 and 2017–2018.

## 1. Introduction

One person using five or more prescription medications simultaneously is commonly defined as polypharmacy [1,2]. While older patients with multimorbidity are more likely to experience polypharmacy [1,3], over the past 20 years, adult prescription drug use and polypharmacy have significantly increased [2], especially in developed nations [4]. Introducing new treatments, studies on drug interactions and side effects, and the increasing need to manage complications are likely reasons for the widespread increase in polypharmacy [5]. Another reason could be that the current guidelines do not adequately address individuals taking multiple medications simultaneously. It has been noted that individuals who experience polypharmacy may be more susceptible to potentially inappropriate medication use [6].

Most evidence indicates that polypharmacy is associated with adverse clinical outcomes, such as hospitalizations, medication–drug interactions, nonadherence to treatment, and adverse drug events (e.g., falls, fractures, and renal failure) [7,8,9]. Moreover, polypharmacy has been connected to higher chances of cognitive decline, disability, and death [5,10]. Furthermore, people with multimorbidity frequently experience polypharmacy, and those with many medications tend to have more unplanned hospitalizations [11], which drives up expenditures for both individuals and healthcare systems [12]. In addition, patients with multiple morbidities who see different providers may be prescribed redundant or interfering therapies because healthcare is typically differentiated without shared records [13].

It has been estimated that medicine errors in hospitals and medication side effects account for approximately 275 and 689 deaths annually in the United States (US), respectively [14]. Furthermore, the alleged increase in the prevalence of polypharmacy in the US from 8.2% in 1999–2000 to 17.1% in 2017–2018 [2], emphasizes the importance of figuring out how sociodemographic factors (such as age, gender, race, and veteran status), socioeconomic status (SES) (such as educational level, marital status, and yearly income), and polypharmacy relate to each other in US individuals. According to prior research, it is commonly believed that characteristics related to polypharmacy include advanced age, gender, race/ethnicity, illiteracy, low physical activity, persistent anger, the use of mobility aids, and the number of diseases [3,15,16].

Although the literature indicates that SES may be a significant factor in polypharmacy [17,18], the significance of SES with rising polypharmacy levels is unclear [19]. For example, the findings of a prior study [20] showed that race/ethnicity, age, marital status, and employment did not correlate with polypharmacy among older Americans, but low income and education levels were linked to increased chances of polypharmacy in this population. According to the findings of another study [18], neither income nor education were linked to polypharmacy in older first-generation Mexican American immigrants to the US. Nonetheless, a recent study [17] on hospitalized patients in Jordan showed that patients with lower monthly incomes experienced less polypharmacy than patients with higher monthly incomes.

Given this inconsistency in the literature, additional research is needed to understand the relationship between sociodemographic factors, SES, and polypharmacy risk. Thus, this study aimed to investigate this relationship in US individuals between 1999–2000 and 2017–2018. This study hypothesized that sociodemographic factors and SES, such as higher education and income, are associated with the risk of polypharmacy because individuals with a higher SES may be more likely to seek medical advice from specialists and, as a result, exhibit greater levels of medication usage [21].

## 2. Materials and Methods

### 2.1. Data Source

This ten-cycle cross-sectional study used National Health and Nutrition Examination Survey (NHANES) data. Since its launch in 1999, NHANES has been a continuous multi-phase nationally representative survey of civilians living in all 50 states and the District of Columbia. NHANES is designed to examine the health status of the US populace. Research visits at mobile examination facilities and in-home interviews were used to gather all NHANES data in 2-year cycles. Data from ten cycles, from 1999–2000 to 2017–2018, were used in this investigation. Data from these years were used because NHANES was launched in 1999 and the data after 2018 are not open for free public use. The overall response rates varied from 52% to 84% and 49% to 80% for both the interview and the examination components, respectively.

The Institutional Review Board (IRB) of the National Center for Health Statistics approved NHANES. Every participant in NHANES signed an informed consent form. This study did not require informed consent or IRB approval because it used secondary data.

### 2.2. Participants

A total of 142,244 participants aged 18 years and older were included from the ten cycles from 1999–2000, 2001–2002, 2003–2004, 2005–2006, 2007–2008, 2009–2010, 2011–2012, 2013–2014, 2015–2016, and 2017–2018. All participants were divided into yes (*n* = 66,932) and no (*n* = 75,305). The yes group consisted of those who used five or more prescription medications (polypharmacy) [2,22]. Participants who used fewer than five medications were referred to as the no group. Pregnant women, those under the age of 18, those with insufficient knowledge, and those who did not know of or refused to report polypharmacy, sociodemographic characteristics, or SES were excluded.

### 2.3. Polypharmacy

Polypharmacy was assessed during the home interview using the computer-assisted personal interview method. The trained interviewer asked participants the following question about prescription needs: “Have you taken any prescription medicines in the past month?” Those who said “yes” were asked to present the interviewer with the prescription containers for each item they had used. The interviewer input the entire product name from the container into a computer for each medicine that was reported. The interviewer requested that the individual state the name of the drug orally if there was no container available.

The Lexicon Plus^®^ (Cerner Multum, Inc., Denver, CO, USA) prescription medication database was modified and uploaded to a laptop computer and incorporated into a search engine for use by the interviewers. This database assists in matching medication names to prescription drug databases to find exact matches or similar text matches. All prescription (and certain over-the-counter) drug products sold in the United States are listed in the extensive Lexicon Plus^®^ database. The interviewer entered the name of a medication and chose the best match from a list of potential matches. For quality control, the products chosen from the computer drug list and originally entered by the interviewer were saved as distinct variables. The interviewer was instructed to choose “drug not found on the list” if the medication could not be located in the database with an exact or comparable match. A previous study used a similar assessment [2].

### 2.4. Sociodemographic Factors and Socioeconomic Status

Age, gender, ethnicity, and veteran status were defined as the self-reported sociodemographic factors of the sample that was included. Educational level, marital status, and yearly income were among the self-reported SES of the sample that was included based on the data related to social determinants of health in NHANES [23]. The sociodemographic factors of all participants were categorized by age (18–44, 45–64, and ≥65 years), gender (men and women), ethnicity (non-Hispanic white, non-Hispanic black, Mexican American, and other), and veteran status (veteran and non-veteran). The classification of SES included educational level (<high school, high school graduates, some college education, college graduate, or above), marital status (married, widowed, divorced, separated, or never married), and annual income in USD (<25,000, 25,000–55,000, and >55,000). A similar classification was used [2,24].

### 2.5. Statistical Analysis

A yes- or no-group stratification was used for the descriptive analysis. Means (*x*) and standard deviations (SDs) were used to display continuous values. A count (n) and an unweighted proportion (%) were displayed for the category variables. The significant difference between groups was computed using the chi-square test, independent Student’s *t*-test, or ANOVA. The change in the unweighted proportion was computed using the following formula: $C=\frac{x2-x1}{x1}\times 100$, where C = relative change, *x*_1_ = initial value, and *x*_2_ = final value.

The relationship between sociodemographic factors, SES, and polypharmacy risk was investigated using multivariable logistic regression adjusted for the same sociodemographic factors and SESs (i.e., age, gender, race/ethnicity, veteran status, educational attainment, married status, and annual income). Age of 18–44 years, men, Mexican Americans, <high school education, never married, income of <USD 25K per year, and non-veterans were used as reference groups. Sensitive analyses were performed with sociodemographic factors and SESs in 1999–2000, 2001–2002, 2003–2004, 2005–2006, 2007–2008, 2009–2010, 2011–2012, 2013–2014, 2015–2016, and 2017–2018. Odds ratios (ORs) and corresponding 95% confidence intervals (CIs) were reported.

Weights were assigned to the interview subsamples of all ten cycles to ensure that the estimates accurately reflected the entire civilian noninstitutionalized US population. We modified the weights to account for the differential probabilities of selection, nonresponse, and noncoverage. Weights and design variables were used to obtain objective estimations and standard errors. A full-case analysis was used if the amount of missing data for the primary analyses was 10% or less. All analyses were conducted using SAS software, version 9.4 (SAS Institute Inc., Cary, NC, USA), with a significance level of less than 0.05.

## 3. Results

**Figure 1:**
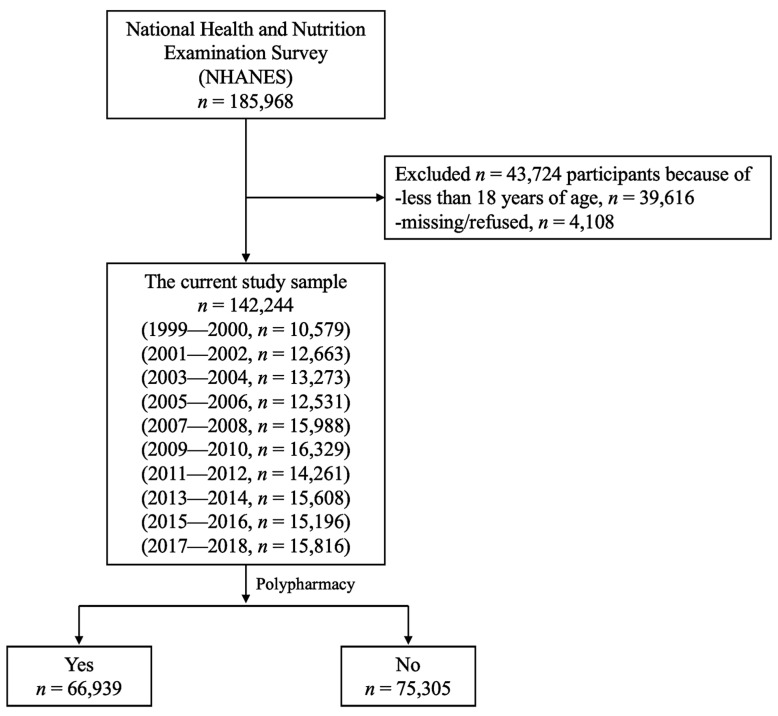
Flowchart of the study participants between 1999–2000 and 2017–2018.

The flow of the study sample is presented in Figure 1. Of 185,968 participants, 142,244 were included in the present study. Individuals aged less than 18 years (*n* = 39,616), missing data, and those who refused to self-report prescription medicines or their characteristics (*n* = 4108) were excluded.

The basic sociodemographic characteristics of participants, including the presence and absence of polypharmacy, are presented in Table 1. Among the 142,244 participants included 66,939 (47.1%) participants with a weighted average age of 65.3 years experienced polypharmacy. Significantly, most individuals experiencing polypharmacy were (*p < 0.0001*) 16 years older on average than individuals not experiencing polypharmacy. A large percentage of people significantly (*p < 0.0001*) experienced polypharmacy, especially those who were 65 years of age or older (57.2%), women (55.1%), and non-Hispanic whites (61.6%). Most persons experiencing polypharmacy were married (52.3%), had a yearly income of less than USD 25,000 (49.6%), and had completed high school (41.4%). 

**Figure 2:**
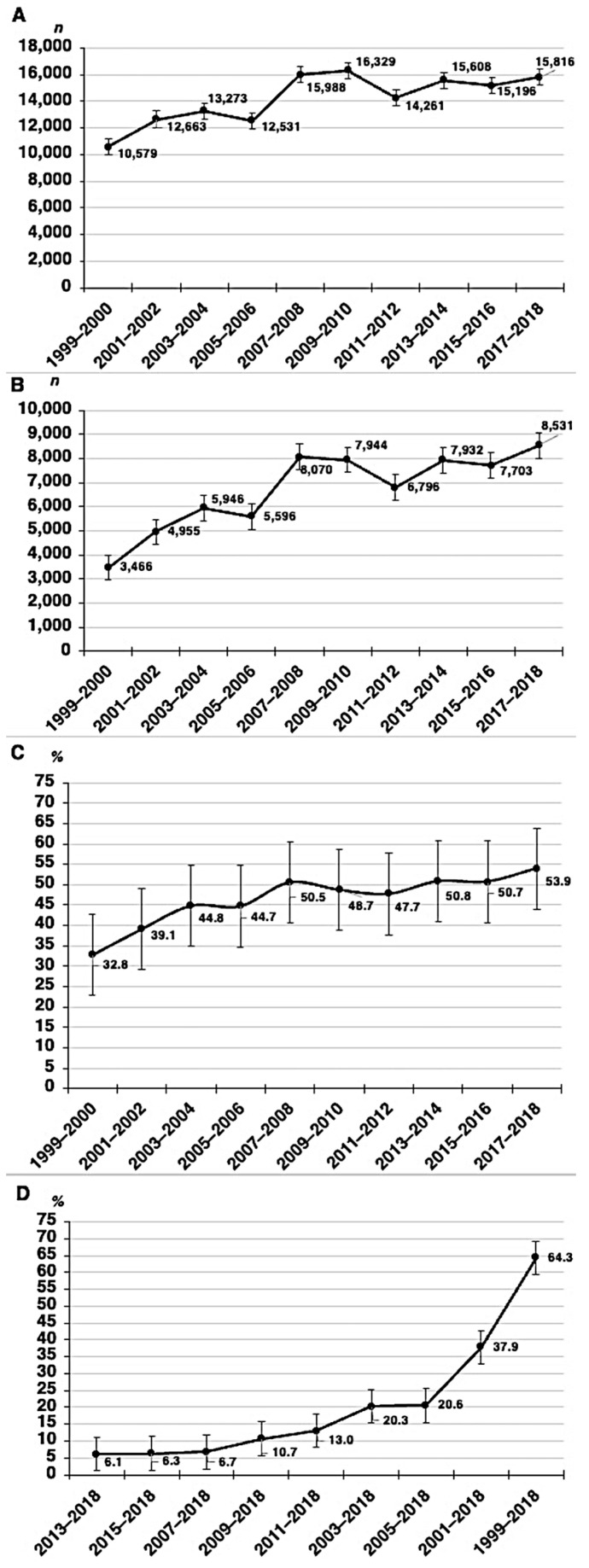
No. of participants (**A**), no. experiencing polypharmacy (**B**), proportion (**C**), and change in proportion (**D**) of polypharmacy, stratified by each 2-year cycle between 1999–2000 and 2017–2018. Polypharmacy is defined as five or more prescription medications taken or used in the past month. Error bars indicate 95% CIs.

The distribution of frequencies, unweighted proportions, and change in unweighted proportions by the 2-year cycles is shown in Figure 2. The unweighted percentage of individuals experiencing polypharmacy significantly increased from 32.8% in 1999–2000 to 53.9% in 2017–2018. The years 2013–2014 (50.8%), 2015–2016 (50.7%), and 2017–2018 (53.9%) showed the greatest percentages. However, there was a greater change in the percentage in 1999–2018 (64.3%), followed by 2001–2018 (37.9%). Table 2 shows the distribution of polypharmacy among US adults by SES in 1999–2000, 2001–2002, 2003–2004, 2005–2006, 2007–2008, 2009–2010, 2011–2012, 2013–2014, 2015–2016, and 2017–2018.

There was a statistically significant association found between polypharmacy and sociodemographic factors between 1999 and 2000 and 2017–2018, such as age between 45 and 64 years (OR = 3.76; 95% CI = 3.60–3.92; *p* < *0.0001*), age of 65 years and above (OR = 3.96; 95% CI = 3.79–4.13; *p* < *0.0001*), women (OR = 1.09; 95% CI = 1.06–1.13; *p* < *0.0001*), non-Hispanic blacks (OR = 1.66; 95% CI = 1.51–1.83; *p* < *0.0001*), being married (OR = 1.14; 95% CI = 1.08–1.19; *p = 0.031*), widowed, divorced, or separated (OR = 1.21; 95% CI = 1.15–1.26; *p* < *0.0001*), and veterans (OR = 1.27; 95% CI = 1.22–1.31; *p* < *0.0001*) (Table 3). 

Table 4 shows the risk of polypharmacy among US adults by sociodemographic factors and SES in 1999–2000, 2001–2002, 2003–2004, 2005–2006, 2007–2008, 2009–2010, 2011–2012, 2013–2014, 2015–2016, and 2017–2018. In 1999–2000, those who were married and had veteran status had 2.41 (95% CI = 1.73–3.40; *p* < *0.0001*) and 1.69 (95% CI = 1.33–2.15; *p* < *0.0001*) times the odds of polypharmacy, respectively. In 2017–2018, those who were aged ≥45 years and had college graduate or above education had 1.91 (95% CI = 1.53–2.38; *p* < *0.0001*) and 2.16 (95% CI = 1.71–2.71; *p* < *0.0001*) times the odds of polypharmacy, respectively.

## 4. Discussion

This study investigated the relationship between sociodemographic factors, SES, and polypharmacy risk in US individuals using ten cycles of nationally representative NHANES data between 1999–2000 and 2017–2018. The findings show that US individuals who were 45 years of age and above, especially women, non-Hispanic black, married, widowed, divorced, or separated, and veterans, were independently significantly associated with polypharmacy risk between 1999–2000 and 2017–2018. The findings also show that there was a shift in the sociodemographic factors and SES linked to polypharmacy risk. In 1999–2000, for instance, the risk of polypharmacy was linked to marriage and veteran status. In contrast, in 2017–2018, persons who were more educated and had higher yearly incomes—especially women and non-Hispanic blacks—were linked to a higher likelihood of polypharmacy.

The results of this study are consistent with those of a recent study [1], which indicated that being older and a woman are risk factors for polypharmacy among older patients in US outpatient settings. Studies [1,25,26] also suggested that the prevalence of polypharmacy among individuals aged 65 years and older remains high. According to a more recent study [27], the older Spanish population had a 23.2% prevalence of polypharmacy between 2011 and 2020. Spanish women (28.1%) exhibited a higher prevalence than men (17.2%). In addition, the study demonstrated that married and widowed Spanish men and women 75 years of age and older had an increased risk of polypharmacy. One potential explanation for the higher likelihood of polypharmacy in older adults could be a health issue that worsens with age and necessitates the number of medications used [28,29].

In line with previous research [30,31], this study found that women were more likely than men to experience polypharmacy. This could be because women more frequently have multiple morbidities, which makes medication use necessary for a longer life expectancy [30]. Another explanation could be that women are more likely to seek help for health conditions than men [32] due to variations in chronic disease patterns, healthcare consumption, and health habits [33]. Similar to our findings that polypharmacy increased in non-Hispanic whites, data from the Medical Expenditure Panel Survey indicated that non-Hispanic whites were more likely than non-Hispanic blacks and Hispanic whites to have access to new medications [34]. There was, however, an additional risk of polypharmacy for non-Hispanic blacks. Non-Hispanic blacks may need to take possibly unsuitable medications due to a greater association between low income and polypharmacy [20,35]. Further research is required to assess the race and ethnic differences in the risk of polypharmacy, as previous studies [20,35] have shown inconsistent results.

According to a prior study [36], married US people were more likely to experience polypharmacy, which is consistent with the results of this study. Nevertheless, because the sample was unrepresentative and consisted of older African Americans who were economically disadvantaged and living in impoverished metropolitan areas (south Los Angeles, CA, USA), the study’s findings cannot be applied to the entire US population. A Brazilian study [16] found that older persons who had separated or divorced were more likely to experience polypharmacy, but the relationship was not statistically significant. The findings of this study regarding the relationship between education level and the risk of polypharmacy were in line with those of another study [37]. According to that study, there was no statistically significant association between a low education level and polypharmacy after adjusting for age, sex, comorbidity, and marital status in this population, although the older Swedish population with lower levels of education had a higher prevalence of polypharmacy.

The results of a recent systematic review and meta-analysis study [19] conflict with the findings of this study regarding education and yearly income. Findings from the meta-analysis demonstrated that socioeconomic disparities exist in polypharmacy in older adults, where those with a lower SES, particularly those with lower levels of education and annual income, have greater probabilities of polypharmacy. The results of the present study, however, are consistent with other researchers’ arguments that individuals with lesser educational backgrounds and incomes may be less likely to seek medical advice from specialists and, as a result, exhibit lower levels of medication usage [21]. Furthermore, although individuals with a higher SES may still experience high levels of multimorbidity, their social standing may contribute to improved condition management, access to better healthcare services [38], and shorter wait times for medical consultations, all of which may affect the number of medications used [39,40]. 

However, the relationship between low SES and access to care is complicated by the complexity of the US healthcare system [41], which varies from state to state. For example, the middle class finds it more difficult to pay for insurance and afford medical treatment according to their income [39]. Furthermore, because of their lower insurance coverage and language barriers, some illegal migrants have difficulties accessing care and medications [42]. According to a review [43], the 38 states (plus Washington) that benefited from Medicaid expansion under the Affordable Care Act saw improvements in coverage, federal receipts, and health outcomes related to access to care. A racial or ethnic minority made up over 56.4% of Medicaid recipients in 2019. However, there were variations in outcomes, quality, and accessibility across these groups.

This study demonstrated that there is a significant risk of polypharmacy among veterans. This could be because many veterans, especially older adults, commonly have many chronic diseases (multimorbidity) [44,45]. The co-occurrence of traumatic brain injury (TBI), chronic pain, and post-traumatic stress disorder (PTSD) is known as the poly-trauma clinical triad and has been applied to many veterans’ illnesses [46]. Although TBI can result in a variety of concomitant ailments, such as burns, amputations, fractures, spinal cord injuries, eye injuries, and auditory trauma, the two most common and functionally incapacitating illnesses may be PTSD and chronic pain. Furthermore, there is a growing epidemic of prescription opioid misuse [47], functional impairment, and depression [48] that coexist with symptoms of chronic pain. PTSD has been linked to poor health, including obesity [49], type-2 diabetes [50], and cardiovascular disorders such as hypertension [51]. Therefore, veterans frequently utilize polypharmacy to control all these symptoms and avoid the development of new medical illnesses as well as the complications of existing diseases. Consequently, it emphasizes the necessity of a single significant initiative that seeks to meet the healthcare requirements of veterans [52]. One valuable initiative is deprescribing (or the deliberate, proactive, and prudent withdrawal of) a medicine that is no longer required or for which the potential risk exceeds the potential benefit [53].

### 4.1. Strengths and Limitations

The main strength of this study is the utilization of ten-cycle big data from a continuous multiphase nationally representative NHANES survey, including veterans and members of many ethnic groups. However, there are still several limitations to the present study. First, there are no causal implications from this study due to its cross-sectional methodology. Further investigation is required to determine whether SES is a causal factor in multiple medicine use. Second, the definition is not comprehensive. The definition of polypharmacy used in this study was based solely on quantity (≥5), as per the literature [22], and did not take into account its potential reasonable use. Since this study did not know the nature of the incorrect use of medications, it simply examined polypharmacy. Furthermore, this study’s findings emerged from patients’ self-reported medication use, which is subject to recall bias. In the future, objective measurements such as insurance data or pharmacy records could be employed to monitor drug use and lessen the impact of recollection bias on outcomes. Third, the type of drugs and any possible negative consequences of polypharmacy were not examined in this study; further research on this topic is needed to better understand the risk of polypharmacy. Lastly, data on clinical characteristics such as multimorbidity and chronic diseases, which are relevant to age and more prevalent in older persons, as well as lifestyle factors such as smoking and body mass index, were not included in this study.

### 4.2. Implications

The results of this study may help in the comprehension of the relationship between sociodemographic characteristics, SES, and polypharmacy by scholars, healthcare providers, and US governments. For older persons, polypharmacy is regarded as a syndrome of harm and a challenge to primary care clinicians in the US [14], as the country has one of the highest medication use rates per capita in the world [54]. Moreover, a recent population bulletin reported that the number of people over the age of 65 is projected to at least double from 46 million to more than 98 million by 2060 [54]. Polypharmacy is especially concerning for older adults due to aging-related factors such as multimorbidity [55], adverse drug effects [56], drug interactions [57], medication non-adherence [58], prescribing cascades, risk of hip fracture and falls [59], use of over-the-counter and complementary medications [60], care transitions [61], and changes in pharmacokinetics [62]. 

Aging individuals, especially older persons, may need to take several drugs to treat their complicated medical conditions, making their treatment difficult to manage. As such, one of the key components of complete geriatric care is making the most of patients’ prescription regimens. One of the major effects of polypharmacy when evaluating an older patient with a new complaint is preventable adverse medication events. Until proven otherwise, this potential should always be considered so that hospitalization risks and prescribing cascades can be avoided. Avoiding many undesirable outcomes, such as falls [7], and lowering healthcare expenditures can be achieved by being aware of specific difficulties connected to polypharmacy, such as a higher risk of hip fractures, falls, and reduced cognitive skills [63,64,65]. The development of policies, programs, and services targeted at lowering polypharmacy and encouraging the population’s rational drug use—especially among adults aged 45 years or above, women, non-Hispanic blacks, and married individuals with high incomes and education levels—may be aided by these findings, which could impact primary healthcare practices in the future, especially in geriatric medicine [66]. According to a study, people who did not experience polypharmacy thought they were in better health than people who did [26]. Therefore, the American Geriatrics Society’s Beers Criteria [67] is widely used by physicians, educators, researchers, and healthcare administrators in the US. They are beneficial for potentially inappropriate medication use in older persons. A multifaceted intervention targeting medication safety as a workable solution to public health issues could benefit from the novel idea of polypharmacy stewardship, which aims to minimize medication-related harm and promote appropriate medication use in older adults [68]. The potential benefits of polypharmacy stewardship in older patients with multimorbidity in a hospital context were demonstrated effectively by an open-label non-randomized feasibility trial conducted in Australia in 2023 [69]. Therefore, it is advisable to consider the application of polypharmacy stewardship in a range of healthcare environments.

## 5. Conclusions

This study aimed to investigate the relationship between sociodemographic factors, SES, and polypharmacy risk in US individuals using ten cycles of nationally representative NHANES data from 1999–2000 to 2017–2018. The results of this study justify concentrating on sociodemographic factors (such as age of 45 years or older, women, non-Hispanic blacks, and veterans) and SES, such as higher educational levels and yearly incomes. Effective drug management should be a primary care physician’s top priority in these populations. It is critical to continuously monitor polypharmacy trends and predictors to create targeted interventions that can enhance the aging population’s quality of life.

## Figures and Tables

**Table 1:** Sociodemographic characteristics stratified by polypharmacy status between 1999–2000 and 2017–2018.

Characteristics	Total *n* (%) ^a^	Polypharmacy		*p*-Value
		Yes ^b^	No ^c^	
		*n* (%) ^a^	*n* (%) ^a^	
Overall	142,244 (100)	66,939 (47.1)	75,305 (52.9)	*<0.0001*
Age in y, *x* ± SD	57.3 ± 16.2	65.3 ± 13.1	49.3 ± 19.3	*<0.0001*
Age group ^d^, y				*<0.0001*
18–44	35,041 (25.3)	5166 (7.8)	29,873 (41.6)	
45–64	45,677 (33.1)	23,215 (35)	22,462 (31.3)	
≥65	57,418 (41.6)	37,994 (57.2)	19,424 (27.1)	
Gender				*<0.0001*
Men	65,628 (46.1)	30,065 (44.9)	35,563 (47.2)	
Women	76,616 (53.9)	36,874 (55.1)	39,742 (52.8)	
Race and ethnicity ^e^				*<0.0001*
Non-Hispanic white	70,336 (57.1)	36,732 (61.6)	33,604 (52.9)	
Non-Hispanic black	20,077 (16.3)	6966 (11.7)	13,111 (20.7)	
Mexican American	30,353 (24.7)	14,656 (24.6)	15,697 (24.7)	
Other	2335 (1.9)	1255 (2.1)	1080 (1.7)	
Education level ^f^				*<0.0001*
<High school	18,876 (13.8)	10,072 (15.1)	8804 (12.5)	
High school graduate	54,258 (39.5)	27,552 (41.4)	26,706 (37.8)	
Some college	38,292 (27.9)	18,681 (28.0)	19,611 (27.8)	
College graduate or above	25,823 (18.8)	10,320 (15.5)	15,503 (21.9)	
Marital status ^g^				*<0.0001*
Married	71,133 (53.9)	33,736 (52.3)	37,397 (55.4)	
Widowed, divorced, or separated	41,818 (31.7)	25,449 (39.5)	16,369 (24.3)	
Never married	19,035 (14.4)	5326 (8.2)	13,709 (20.3)	
Annual income ^h^				*<0.0001*
<USD 25K	47,366 (43.6)	26,471 (49.6)	20,895 (37.7)	
USD 25K–55K	40,150 (36.9)	18,794 (35.2)	21,356 (38.6)	
>USD 55K	21,180 (19.5)	8080 (15.2)	13,100 (23.7)	
Veteran status ^i^				*<0.0001*
Veteran	23,153 (16.3)	14,242 (21.3)	8911 (11.8)	
Non-veteran	119,023 (83.7)	52,663 (78.7)	66,360 (88.2)	

**^a^** Weighted sample size and the number of cases. The total number of participants was 142,244 from the interview sample. **^b^** Five or more prescription medications taken or used in the past month. **^c^** Less than five prescription medications taken or used in the past month. **^d^** Participants (*n* = 4108) refused to report or did not know their age. **^e^** Race and ethnicity were determined by self-report in fixed categories. The “other” group included other non-Hispanic races or multiple races. Participants (*n* = 19,143) refused to report or did not know their race/ethnicity. **^f^** Participants (*n* = 4995) refused to report or did not know their education level. **^g^** Participants (*n* = 10,258) refused to report their marital status. **^h^** Participants (*n* = 33,548) refused to report their income level. **^i^** Participants (*n* = 68) refused to report their veteran status.

**Table 2:** Distribution of polypharmacy by socioeconomic status between 1999–2000 and 2017–2018.

Characteristics	Polypharmacy in Adults ^a^, % (95% CI)									
	1999–2000 (*n* = 10,579)	2001–2002 (*n* = 12,663)	2003–2004 (*n* = 13,273)	2005–2006 (*n* = 12,531)	2007–2008 (*n* = 15,988)	2009–2010 (*n* = 16,329)	2011–2012 (*n* = 14,261)	2013–2014 (*n* = 15,608)	2015–2016 (*n* = 15,196)	2017–2018 (*n* = 15,816)
Age group, y										
18–44	1.8 (1.6, 2.0)	3.9 (3.5, 4.2)	3.6 (3.3, 3.9)	4.3 (3.9, 4.6)	3.7 (3.4, 4.0)	3.4 (3.1, 3.7)	4.5 (4.1, 4.9)	4.2 (3.9, 4.5)	3.6 (3.3, 3.9)	3.7 (3.4, 4.0)
45–64	11.4 (10.8, 12.1)	12.5 (11.9, 13.2)	14.3 (13.6, 14.9)	16.0 (15.3, 16.7)	18.7 (18.1, 19.3)	17.3 (16.7, 17.9)	17.3 (16.7, 18.0)	20.8 (20.1, 21.4)	18.5 (17.9, 19.2)	17.6 (17.1, 18.2)
≥65	20.4 (19.7, 21.6)	23.9 (23.1, 24.6)	28.2 (27.4, 28.9)	25.9 (25.1, 26.7)	28.9 (28.1, 29.6)	28.6 (27.9, 29.3)	26.5 (25.9, 27.3)	26.7 (26.0, 27.4)	29.0 (28.3, 29.8)	33.1 (32.3, 33.8)
Sex										
Men	13.4 (12.7, 14.0)	16.8 (16.1, 17.4)	19.8 (19.1, 20.4)	19.6 (18.5, 20.3)	22.3 (21.7, 23.0)	22.1 (21.5, 22.8)	21.6 (20.9, 22.3)	21.5 (20.8, 22.1)	24.2 (23.5, 24.8)	26.0 (25.3, 26.3)
Women	19.3 (18.6, 20.1)	22.3 (21.6, 23.1)	25.0 (24.2, 25.8)	25.0 (24.2, 25.7)	28.1 (27.3, 28.8)	26.4 (25.8, 27.1)	26.0 (25.3, 26.7)	29.3 (28.6, 30.0)	26.4 (25.8, 27.1)	27.9 (27.2, 28.6)
Race and ethnicity ^b^										
Non-Hispanic white	18.0 (17.3, 18.8)	27.2 (26.4, 28.0)	31.4 (30.6, 32.2)	28.5 (27.7, 29.3)	30.4 (29.7, 31.1)	28.6 (27.9, 29.3)	26.3 (25.5, 27.0)	30.8 (30.0, 31.6)	25.0 (24.2, 25.7)	30.5 (29.7, 31.3)
Non-Hispanic black	7.1 (6.6, 7.6)	7.1 (6.6, 7.5)	7.0 (6.6, 7.5)	11.7 (11.1, 12.3)	11.2 (10.7, 11.7)	9.2 (8.8, 9.6)	16.1 (15.4, 16.8)	12.9 (12.3, 13.4)	12.6 (12.0, 13.1)	15.7 (15.0, 16.3)
Mexican American	2.2 (2.0, 2.6)	0.8 (0.6, 0.9)	0.6 (0.4, 0.7)	0.3 (0.2, 0.4)	4.6 (4.2, 4.9)	4.6 (4.2, 4.9)	4.5 (4.1, 4.9)	3.2 (2.9, 3.5)	6.6 (6.2, 7.0)	4.9 (4.5, 5.3)
Other	5.1 (4.7, 5.5)	4.2 (3.9, 4.6)	5.9 (5.5, 6.4)	4.2 (3.8, 4.5)	4.6 (4.3, 5.0)	6.5 (6.1, 6.9)	3.3 (2.9, 3.6)	5.4 (5.1, 5.8)	7.8 (7.3, 8.2)	5.3 (4.9, 5.7)
Education level										
<High school	8.2 (7.7, 8.8)	6.7 (6.2, 7.1)	8.3 (7.8, 8.7)	6.6 (6.2, 7.1)	9.2 (8.8, 9.7)	7.8 (7.4, 8.2)	7.1 (6.7, 7.6)	5.1 (4.8, 5.5)	8.4 (7.9, 8.8)	5.7 (5.3, 6.1)
High school graduate	15.5 (14.8, 16.2)	16.9 (16.2, 17.5)	20.5 (19.8, 21.1)	20.0 (19.3, 20.7)	22.6 (21.9, 23.3)	21.3 (20.7, 21.9)	19.2 (18.5, 19.8)	21.4 (20.7, 22.0)	18.6 (18.0, 19.2)	22.0 (21.3, 22.6)
Some college	7.7 (7.1, 8.2)	10.2 (9.6, 10.7)	12.2 (11.7, 12.8)	12.7 (12.1, 13.3)	12.3 (11.8, 12.8)	14.5 (14.0, 15.1)	14.3 (13.7, 14.9)	15.6 (15.0, 16.2)	15.7 (15.0, 16.2)	17.5 (16.9, 18.1)
College graduate or above	3.0 (2.7, 3.3)	7.1 (6.7, 7.6)	6.0 (5.5, 6.4)	7.6 (7.1, 8.1)	7.2 (6.8, 7.6)	5.8 (5.4, 6.1)	8.0 (7.6, 8.5)	9.8 (9.4, 10.3)	8.9 (8.4, 9.3)	9.7 (9.1, 10.1)
Marital status										
Married	18.8 (17.9, 19.5)	21.3 (20.6, 22.0)	23.8 (23.0, 24.6)	23.7 (22.9, 24.5)	28.1 (27.3, 28.9)	27.3 (26.6, 28.1)	23.9 (23.2, 24.7)	28.1 (27.4, 28.9)	26.8 (26.0, 27.5)	29.5 (28.7, 30.2)
Widowed, divorced, or separated	12.3 (11.7, 13.0)	16.5 (15.9, 17.2)	19.4 (18.8, 20.1)	18.9 (18.2, 19.6)	20.0 (19.4, 20.7)	19.2 (18.6, 19.9)	20.8 (20.1 21.5)	20.1 (19.4, 20.8)	20.8 (20.1, 21.5)	21.5 (20.8, 22.1)
Never married	2.4 (2.1, 2.7)	1.9 (1.7, 2.2)	2.4 (2.2, 2.7)	3.2 (2.9, 3.5)	4.4 (4.1, 4.7)	4.3 (4.0, 4.6)	5.1 (4.7, 5.4)	4.9 (4.5, 5.2)	5.1 (4.7–5.5)	5.2 (4.8, 5.6)
Annual income										
<USD 25K	19.7 (18.8, 20.5)	17.0 (16.3, 17.7)	21.2 (20.4, 21.9)	19.0 (18.2, 19.6)	28.6 (27.8, 29.5)	25.9 (25.1, 26.7)	29.1 (28.2, 30.0)	28.3 (27.5, 29.2)	28.9 (27.9, 29.7)	26.1 (25.2, 26.9)
USD 25K–55K	8.2 (7.6, 8.8)	11.9 (11.3, 12.5)	14.5 (13.9, 15.2)	15.4 (14.8, 16.0)	20.9 (20.2, 21.6)	20.7 (19.9, 21.3)	16.5 (15.7, 17.2)	20.0 (19.3, 20.8)	18.9 (18.2, 19.7)	24.2 (23.4, 25.1)
>USD 55K	5.0 (4.5, 5.4)	10.3 (9.8, 10.9)	9.5 (9.0, 10.0)	10.0 (9.5, 10.6)	5.0 (4.6, 5.3)	5.4 (5.0, 5.8)	5.9 (5.4, 6.3)	7.3 (6.8, 7.8)	6.7 (6.2, 7.2)	8.0 (7.4, 8.5)
Veteran status										
Veteran	7.0 (6.6, 7.5)	9.1 (8.6, 9.6)	11.9 (11.4, 12.5)	11.7 (11.1, 12.2)	10.8 (10.3, 11.3)	11.1 (10.7, 11.6)	8.8 (8.3, 9.2)	8.7 (8.2, 9.1)	9.6 (9.1, 10.1)	10.5 (10.0, 11.0)
Non-veteran	25.5 (24.7, 26.3)	29.9 (29.2, 30.7)	32.8 (32.0, 33.6)	32.8 (32.0, 33.6)	39.7 (38.9, 40.4)	37.5 (36.7, 38.2)	38.9 (38.1, 39.7)	42.1 (41.3, 42.9)	41.0 (40.3, 41.8)	43.4 (42.6, 44.1)

Abbreviations: US—United States; USD—United States dollar; CI—confidence interval; y—year. **^a^** The definition of polypharmacy is consistent across all years. All estimates are age-standardized to the 2017–2018 National Health and Nutrition Examination Survey nonpregnant adult population, using the age groups 18–44 years, 45–64 years, and 65 years or older. **^b^** Race and ethnicity are determined by self-report in fixed categories. The “other” group includes other non-Hispanic races or multiple races. Participants (*n* = 387) who refused to report their race or ethnicity.

**Table 3:** Association between sociodemographic factors, socioeconomic status, and polypharmacy risk in all ten cycles between 1999–2000 and 2017–2018.

Characteristics	Polypharmacy in Adults ^a^	
	Adjusted ^b^ OR (95% CI)	*p*-Value
Sociodemographic factors		
18–44 years	1.00 Ref.	
45–64 years	3.76 (3.60–3.92)	*<0.0001*
≥65 years	3.96 (3.79–4.13)	*<0.0001*
Men	1.00 Ref.	
Women	1.09 (1.06–1.13)	*<0.0001*
Mexican American	1.00 Ref.	
Non-Hispanic white	0.67 (0.64–0.70)	*0.359*
Non-Hispanic black	1.66 (1.51–1.83)	*<0.0001*
Other	1.02 (0.98–1.05)	*0.289*
Non-veteran	1.00 Ref.	
Veteran	1.27 (1.22–1.31)	*<0.0001*
Socioeconomic status		
<High school	1.00 Ref.	
High school graduate	1.01 (0.97–1.06)	*0.0002*
Some college	1.08 (1.03–1.13)	*<0.0001*
College graduate or above	1.21 (1.15–1.27)	*<0.0001*
Never married	1.00 Ref.	
Married	1.14 (1.08–1.19)	*0.031*
Widowed/divorced/separated	1.21 (1.15–1.26)	*<0.0001*
<USD 25K/year	1.00 Ref.	
USD 25K–<55K/year	1.38 (1.34–1.42)	*0.031*
>USD 55K/year	1.86 (1.79–1.93)	*<0.0001*

Abbreviations: ref.—reference; US—United States; USD—United States dollar; CI—confidence interval; K—thousands. **^a^** The definition of polypharmacy is consistent across all years. **^b^** Adjusted for age, gender, race/ethnicity, education level, marital status, annual income, and veteran status.

**Table 4:** Association between sociodemographic factors, SES, and polypharmacy risk, stratified by 2-year cycle between 1999–2000 and 2017–2018.

Characteristics	Polypharmacy in Adults ^a^, Adjusted ^b^ OR (95% CI)									
	1999–2000 (*n* = 2266)	2001–2002 (*n* = 4179)	2003–2004 (*n* = 5258)	2005–2006 (*n* = 4845)	2007–2008 (*n* = 6219)	2009–2010 (*n* = 5504)	2011–2012 (*n* = 4572)	2013–2014 (*n* = 5515)	2015–2016 (*n* = 4703)	2017–2018 (*n* = 5010)
Sociodemographic factors										
18–44 years, ref.	1.00	1.00	1.00	1.00	1.00	1.00	1.00	1.00	1.00	1.00
45–64 years	1.07 (0.90, 1.26)	1.09 (0.97, 1.23)	1.62 (1.31, 199) *	1.92 (1.57, 2.35) *	1.61 (1.33, 1.94) *	1.21 (0.99, 1.48)	1.25 (1.03, 1.54) **	1.89 (1.58, 2.27) *	1.52 (1.22, 1.90) **	1.91 (1.53, 2.38) *
≥65 years	1.01 (0.68, 1.50)	1.08 (0.87, 1.34)	1.28 (1.05, 1.57) ***	1.28 (1.05, 1.58) ***	1.03 (0.85, 1.24)	1.07 (0.87, 1.29)	1.08 (0.88, 1.32)	1.24 (1.04, 1.49) **	1.41 (1.13, 1.76) ***	1.57 (1.27, 1.95) ***
Men, ref.	1.00	1.00	1.00	1.00	1.00	1.00	1.00	1.00	1.00	1.00
Women	1.94 (1.58, 2.38) *	1.13 (0.98, 1.29)	1.04 (0.91, 1.18)	1.07 (0.93, 1.23)	1.14 (1.02, 1.27) **	1.28 (1.13, 1.44) *	1.40 (1.23, 1.58) *	1.12 (1.00, 1.25) ***	1.02 (0.90, 1.14)	1.28 (1.14, 1.44) *
Mexican American, ref.	1.00	1.00	1.00	1.00	1.00	1.00	1.00	1.00	1.00	1.00
Non-Hispanic white	1.26 (0.96, 1.64)	1.24 (0.99, 1.51)	1.03 (0.90, 1.18)	1.10 (0.98, 1.24)	1.32 (1.19, 148) *	1.11 (0.98, 1.25)	1.15 (1.01, 1.29) **	1.20 (1.07, 1.34) ***	1.75 (1.54, 1.99) *	1.08 (0.95, 1.21)
Non-Hispanic black	1.73 (1.27, 2.36) **	1.85 (1.47, 2.32) *	1.43 (0.94, 2.19)	2.61 (1.42, 4.81) *	1.76 (1.49, 2.09) *	1.09 (0.90, 1.25)	1.16 (0.95, 1.39)	1.58 (1.30, 1.92) **	1.06 (0.89, 1.25)	1.23 (1.03, 1.49) **
Other	1.13 (0.90, 1.44)	1.05 (0.87, 1.27)	1.73 (1.48, 2.01) *	2.82 (2.34, 3.39) *	1.53 (1.29, 1.80) *	1.15 (0.99, 1.34)	1.02 (0.84, 1.25)	1.40 (1.18, 1.66) ***	1.50 (1.28, 1.76) **	1.19 (0.99, 1.43)
Non-veteran, ref.	1.00	1.00	1.00	1.00	1.00	1.00	1.00	1.00	1.00	1.00
Veteran	1.69 (1.33, 2.15) *	1.11 (0.94, 1.30)	1.11 (0.96, 1.28)	1.07 (0.82, 1.24)	1.53 (1.33, 1.74) *	1.68 (1.45, 1.94) *	1.33 (1.13, 1.56) **	1.29 (1.12, 1.49) **	1.49 (1.29, 1.74) *	1.03 (0.89, 1.19)
Socioeconomic status										
<High school education, ref.	1.00	1.00	1.00	1.00	1.00	1.00	1.00	1.00	1.00	1.00
High school graduate	1.59 (1.31, 1.95) *	1.01 (0.88, 1.15)	1.11 (0.96, 1.29)	1.43 (1.22, 1.67) ***	1.11 (0.98, 1.26)	1.64 (1.41, 1.90) *	1.07 (0.92, 1.25)	1.08 (0.93, 1.33)	1.09 (0.92, 1.27)	1.34 (1.11, 1.61) ***
Some college	1.12 (1.10, 1.36) ***	1.09 (0.95, 1.27)	1.06 (0.90, 1.25)	1.45 (1.21, 1.67) ***	1.20 (1.04, 1.39) ***	1.08 (0.92, 1.27)	1.07 (0.89, 1.27)	1.11 (0.93, 1.33)	1.26 (1.07, 1.49) *	1.29 (1.07, 1.56) ***
College graduate or above	1.13 (0.82, 1.57)	1.34 (0.98, 1.82)	1.00 (0.83, 1.21)	1.44 (1.18, 1.75) ***	1.26 (1.05, 1.52) *	1.53 (1.24, 1.89) **	1.14 (0.92, 1.41)	1.36 (1.10, 1.68) *	1.03 (0.84, 1.26)	2.16 (1.71, 2.71) *
Never married status, ref.	1.00	1.00	1.00	1.00	1.00	1.00	1.00	1.00	1.00	1.00
Married	2.41 (1.71, 3.40) *	1.36 (1.02, 1.81) ***	1.06 (0.83, 1.34)	1.56 (1.25, 1.93) **	1.42 (1.21, 1.67) *	1.35 (1.13, 1.61) *	1.01 (0.83, 1.24)	1.01 (0.86, 1.19)	1.44 (1.19, 1.75) ***	1.09 (0.91, 1.30)
Widowed/divorced/separated	1.32 (0.93, 1.87)	1.29 (0.97, 1.73)	1.01 (0.79, 1.29)	1.51 (1.21, 1.88) **	1.26 (1.07, 1.49) ***	1.04 (0.87, 1.25)	1.16 (0.94, 1.41)	1.06 (0.89, 1.25)	1.65 (1.37, 2.00) *	1.18 (0.99, 1.41)
<USD 25K per year, ref.	1.00	1.00	1.00	1.00	1.00	1.00	1.00	1.00	1.00	1.00
USD 25K–<55K /year	1.40 (1.15, 1.69) *	1.02 (0.89, 1.17)	1.08 (0.96, 1.21)	1.22 (1.08, 1.38) ***	1.48 (1.34, 1.64) ***	1.64 (1.48, 1.82) ***	1.51 (1.34, 1.69) *	1.33 (1.19, 1.48) ***	1.36 (1.21, 1.53) ***	1.44 (1.29, 1.60) *
>USD 55K /year	1.92 (1.52, 2.43) *	1.01 (0.87, 1.17)	1.79 (1.56, 2.06) *	1.73 (1.49, 2.01) *	2.00 (1.69, 2.37) *	2.61 (2.20, 3.09) *	1.02 (0.85, 1.23)	1.54 (1.32, 1.80) *	1.41 (1.17, 1.68) ***	1.15 (0.98, 1.34)

Abbreviations: ref.—reference; US—United States; USD—United States dollar; CI—confidence interval; K—thousands. * *p* < 0.0001; ** *p =* 0.0001; *** *p* < 0.05. ^a^ The definition of polypharmacy is consistent across all years. All estimates are age-standardized to the 2017–2018 National Health and Nutrition Examination Survey nonpregnant adult population, using the age groups 18–44 years, 45–64 years, and 65 years or older. ^b^ Adjusted for age, gender, race/ethnicity, education level, marital status, annual income, and veteran status.

## Data Availability

Publicly available datasets were analyzed in this study. These data can be found here: https://www.cdc.gov/nchs/nhanes/index.htm. [accessed on 11 January 2024].
